# A Clinical Decision Support System for Predicting Invasive Breast Cancer Recurrence: Preliminary Results

**DOI:** 10.3389/fonc.2021.576007

**Published:** 2021-03-11

**Authors:** Raffaella Massafra, Agnese Latorre, Annarita Fanizzi, Roberto Bellotti, Vittorio Didonna, Francesco Giotta, Daniele La Forgia, Annalisa Nardone, Maria Pastena, Cosmo Maurizio Ressa, Lucia Rinaldi, Anna Orsola Maria Russo, Pasquale Tamborra, Sabina Tangaro, Alfredo Zito, Vito Lorusso

**Affiliations:** ^1^Struttura Semplice Dipartimentale di Fisica Sanitaria, IRCCS Istituto Tumori “Giovanni Paolo II”, Bari, Italy; ^2^Unitá Opertiva Complessa di Oncologia Medica, IRCCS Istituto Tumori “Giovanni Paolo II”, Bari, Italy; ^3^Dipartimento di Fisica, Universitá degli Studi “Aldo Moro” e Istituto Nazionale di Fisica Nucleare - Sezione di Bari, Bari, Italy; ^4^Struttura Semplice Dipartimentale di Radiologia Senologica, IRCCS Istituto Tumori “Giovanni Paolo II”, Bari, Italy; ^5^Unitá Opertiva Complessa di Radioterapia, IRCCS Istituto Tumori “Giovanni Paolo II”, Bari, Italy; ^6^Unitá Opertiva Complessa di Anatomia Patologica, IRCCS Istituto Tumori “Giovanni Paolo II”, Bari, Italy; ^7^Unitá Opertiva Complessa di Chirurgia Plastica e Ricostruttiva, IRCCS Istituto Tumori “Giovanni Paolo II”, Bari, Italy; ^8^Struttura Semplice Dipartimentale di Oncologia Per la Presa in Carico Globale del Paziente, IRCCS Istituto Tumori “Giovanni Paolo II”, Bari, Italy; ^9^Dipartimento di Oncologia Medica, Universitá degli Studi di Napoli, Napoli, Italy; ^10^Dipartimento di Scienze del Suolo, della Pianta e degli Alimenti, Universitá degli Studi “Aldo Moro” e Istituto Nazionale di Fisica Nucleare - Sezione di Bari, Bari, Italy

**Keywords:** invasive breast cancer, cancer recurrence, late recurrence, feature importance, machine learning, prognosis

## Abstract

The mortality associated to breast cancer is in many cases related to metastasization and recurrence. Personalized treatment strategies are critical for the outcomes improvement of BC patients and the Clinical Decision Support Systems can have an important role in medical practice. In this paper, we present the preliminary results of a prediction model of the Breast Cancer Recurrence (BCR) within five and ten years after diagnosis. The main breast cancer-related and treatment-related features of 256 patients referred to Istituto Tumori “Giovanni Paolo II” of Bari (Italy) were used to train machine learning algorithms at the-state-of-the-art. Firstly, we implemented several feature importance techniques and then we evaluated the prediction performances of BCR within 5 and 10 years after the first diagnosis by means different classifiers. By using a small number of features, the models reached highly performing results both with reference to the BCR within 5 years and within 10 years with an accuracy of 77.50% and 80.39% and a sensitivity of 92.31% and 95.83% respectively, in the hold-out sample test. Despite validation studies are needed on larger samples, our results are promising for the development of a reliable prognostic supporting tool for clinicians in the definition of personalized treatment plans.

## Introduction

Breast cancer (BC) is the second most frequently diagnosed cancer and the fifth cause of cancer mortality worldwide, responsible for 6.6% of all deaths (1). The mortality associated to this pathology is in many cases related to metastasization (2)

and recurrence (or relapse) (3). Relapse is documented in 10–15% of all patients (4) and, moreover, it has been proven to be a relevant prognostic factor and a key indicator of BC behavior, intrinsically related to mortality. Early BC is typically treated by surgery, chemotherapy, biological and hormonal therapies, and radiotherapy (5) according to tumor biology and clinical stage of disease, with a curative intent. However, local or distant relapse can occur at any time. Recurrence free survival is the length of time in which the patient remains without signs or symptoms of cancer from the start of the primary curative treatment. It reflects the efficacy of an oncological therapy and it is used as a treatment quality measure. A significant topic in medical research is the improvement of the therapeutic efficacy of BC treatments and thus the recurrence free survival time.

Personalized treatment strategies are critical for the improvement of the BC patients follow-up. An important step has been made in medical practice through Clinical Decision Support Systems (CDSSs). They are computerized systems that analyze several data to support clinical decision-making by improving its quality. However, there are differences in how particular CDSSs are developed, in how patients’ data are included and in the way they are used in clinic. Defining data classification techniques and relative prediction models take on an important role to enhance the reliability of the decision-making system in the breast cancer recurrence (BCR) prognostic prediction. Recent revisions of published works haveproved that achieving a representative data-set for BCR is an arduous task (6, 7). Furthermore no agreement was reached about the most appropriate set of predictors for this disease. In literature, studies with the aim to predict cancer prognosis are limited and, mostly about BCR, so this is still an open question.

High accuracy results are often reached, but sometimes sensitivity is compromised. Moreover, there are often missing data in the patient records which needs an appropriate pre-processing before its use to train a machine learning algorithm. Nonetheless, the mixture of different machine learning techniques together with the identification of suitable features predictive of BCR appears to be the key to achieve better results.

The main state-of-the-art studies use different kinds features mainly attributable to patients’ characteristics (such as age at diagnosis, menarche age, number of children and familiarity for cancer), tumor histopathological characteristics (such as tumorsize, number of involved and dissected lymph nodes, receptor status, histological grade, etc.), type of surgery and oncological treatment performed (chemotherapy, hormone therapy, biological therapies and radiotherapy) (8–11).

The general goal of our research was to develop a reliable CDSS for physicians to predict the probability of disease recurrence after a BC (12).

In this paper, we used histopathological, and therapeutic information of subjects who have had BC and for whom therapeutic follow-up is known at 5 and 10 years after the cancer diagnosis. In order to evaluate the usefulness of collected data, firstly we developed different feature importance techniques, then we evaluated the performance of the most important features subset by training different classifiers at state-of-the-art.

## Materials and Methods

### Materials

#### Inclusion and Exclusion Criteria

For this study, we have considered women referred to *I.R.C.C.S. Istituto Tumori “Giovanni Paolo II”* of Bari (Italy).

Patients were included in the study if:

- they had a first early invasive BC,
- they were disease-free for at least 10 years or presented a disease recurrence within 10 years,
- they were not metastatic ab initio.

Disease recurrence was defined as local recurrence, or the appearance of distant visceral and soft tissue metastases. Patients who developed a controlateral invasive BC or a second malignancy in other organs, and patients who had undergone primary chemotherapy for BC were excluded from the study. In this first phase of the study, we preferred not to include patients treated with primary chemotherapy to obtain a homogeneous sample and to avoid any interference in the analysis of the same chemotherapy. In fact, it would have required the insertion and analysis of many other additional information relating to the characteristics of the tumor after surgery, the therapy itself, its course and success. This would require a much larger sample of patients to obtain meaningful results and a dedicated analysis before being integrated into a generalized mode.

#### Experimental Dataset

A total of 256 patients were collected. Together with our medical oncologists, we have identified the main potentially informative features in order to predict BCR.

Data were collected from the patients’ medical records of our Institute. In addition to the age at the first tumor diagnosis, in the analysis we included the main breast cancer-related and treatment-related factors predictive of recurrence for each patient: Histological subtype (HI: ductal, lobular, other), Carcinoma In Situ associated with invasive component (CIS: present/absent), Vascular Invasion (VI: Absent, Focal, Extended, Present but not typed), Estrogen Receptor expression (ER,% value), Progesterone Receptor expression (PR,% value), cellular marker for proliferation (Ki67,% value), epidermal growth factor receptor-2 (Her2: negative/positive), histological Grade (G, Elston-Ellis scale: 1, 2,3), Tumor size stage (T: staging system classify), lymph Nodes stage (N, staging system classify), Number of Positive lymph nodes (NP), type of Surgery (S: Quadrantectomy/Mastectomy), ChemioTherapy (CT: Yes/No), Trastuzumab Therapy (TT: Yes/No), Hormone Therapy (HT: Yes/No). Therefore 16 features were collected for each patient. Our Institute’s Scientific Board approved the retrospective observational study.

### Methods

We have developed two different models, one to predict BCR within 5 and one within 10 years after the first diagnosis.

For this purpose, we have developed a feature selection process by identified the most stable ones in three different feature importance techniques, that is, those features that more than others seem to make a real contribution to the classification problem regardless of the training sample. After defining a ranking of features collected, we have evaluated the recurrence prediction performances by training three classifiers known to the state-of-the-art on the best features subset. Specifically, the performance of the models was assessed using the hold-out method where the dataset was split into two randomly exclusive sets (80% training and 20% test sets). 100 10-fold cross-validation rounds were used to identified the best features subset and evaluate the best classification models on hold out training set; therefore, the data of the same patient cannot be in training and testing data-set simultaneously. Moreover, we have tested also the best features subset on the other sample (13).

**Figure 1:**
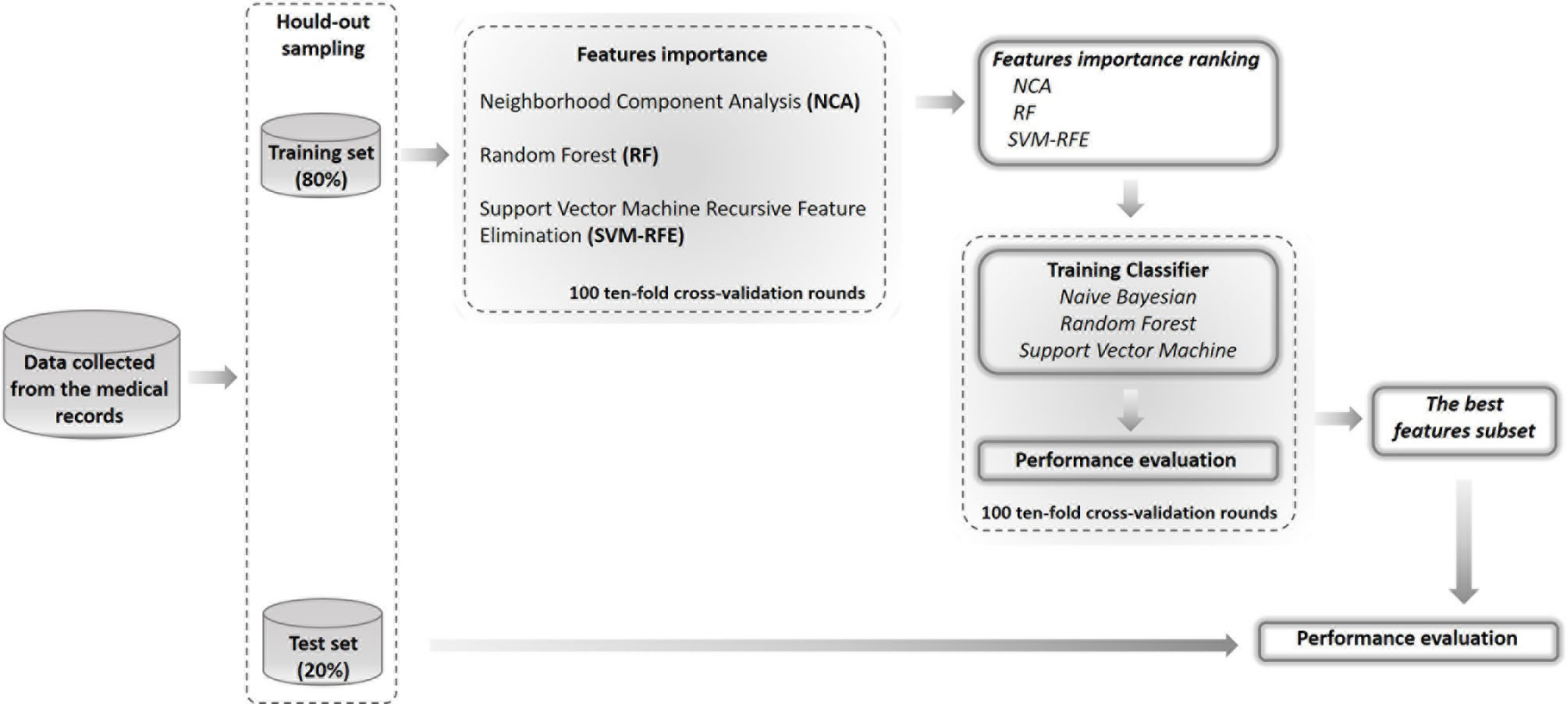
Flow-chart of the proposed model. The performance of the models was assessed by using the hold-out method where the dataset was split into two randomly exclusive sets (80% training and 20% test sets). On training sample of hold-out sampling, we have evaluated the important of features by means three different techniques and identified the best features subset by compering the performances of three different classifiers on 100 10-fold cross-validation rounds. Then, we have tested the best features subset on the test set of hold-out sampling.

In **Figure 1**, a schematic overview of our analysis approach is shown.

In our study, among 256 patients, 162 subjects had complete information (14).

In our work, missing clinical attribute values were treated according to the predictive value imputation method by replacing missing values with the average of the attribute observed in the training set (15).

Our dataset consisted of both categorical and continuous variables. Despite the problem of combining both categorical and continuous data, usually referred to as “mixed data”, in learning algorithms is well known in literature (16) and usually requires sophisticated and dedicated strategies (17). However, it is known that some algorithms, like the ones used in this work, can manage these data (for several purposes) using simple numeric encoding (18–21).

Feature importance techniques and classification models were performed using the MATLAB R2018a (Mathworks, Inc.,Natick, MA, USA) software.

#### Feature Importance Techniques

On the training set of hold-out validation sampling, we have used three different feature importance techniques, such as Neighborhood Component Analysis (NCA), Random Forest (RF) and Support Vector Machine Recursive Feature Elimination (SVM-RFE).

NCA is a non-parametric method to select features with the goal of maximizing prediction accuracy of classification algorithms; the algorithm estimates the feature weights by using a diagonal adaptation of neighborhood component analysis (22, 23).

RF is a know popular machine learning algorithm provide good predictive performance and low over-fitting (24, 25). It provides a measure of features importance ranking them based on how well they improve the purity of the node represented by the single feature; indeed, at each node the algorithm identifies a feature cut-off value that optimizes the division of the dataset into the real classes of belonging. In our work, the impurity measure used was Gini’s diversity index calculated by permuting out-of-bag observations among the trees.

SVM-RFE is a feature importance algorithm that filters relevant features and remove relatively insignificant feature variables in order to achieve higher classification performance (26, 27). SVM-RFE is an iterative procedure of backward feature elimination which utilizes the cost function as the ranking criterion. In particular, firstly, the SVM classifier is trained using training objects to optimize the weights with respect to cost function defined; then, all features are ranked using the weight calculated by the SVM classifier, and finally, the feature with the smallest criterion was eliminated at each iterative step to generate the ranking list of all features.

For each considered techniques, we have calculated the final feature ranking as average of the scores of importance calculating on 100 10-fold cross-validation.

#### Performance Evaluation

Three different state-of-the-art machine learning classifiers, such as Naive Bayesian, RF, and SVM were trained to solve the binary discrimination problem (recurrence vs. control cases) by selecting an increasing features number previously sorted by the average importance score.

Naive Bayes classifier is a probabilistic machine learning model that is used for classification task based on the Bayes theorem (28). They require a small amount of training data to estimate the necessary parameters; despite their apparently over-simplified assumptions, Naive Bayes classifiers worked well in many real-world situations.

RF classifier is robustness against over-fitting and is easy to tune as it depends only on two parameters such as the number of trees to be grown and the number of features to pick at each node split. Therefore, it was a good choice for an exploratory analysis. A standard configuration of RF was adopted with 100 trees and 20 features [as described in Breiman (24)] randomly selected at each split because more complicated architectures did not give any significant classification improvement. Moreover, in order to control the over-fitting risk, we have fixed a small number of observations per tree leaf, such as 5.

SVM classification algorithm estimates a hyperplane separating points in a high dimensional space, so that the examples of the categories are divided by a clear gap that is as wide as possible; new examples are then mapped into that same space and are predicted to belong to a category based on the side of the gap on which they fall (29). In our study, the radial basis function kernels and standard configuration of SVM were used.

The three classifiers considered are characterized by a different approach to solving the classification model. In fact, while Naive Bayesian classifier has a probabilistic approach to classification problem, RF classifier is an ensemble technique, i.e. it combines many decision trees in a single model, so that the forecasts made by decision trees, which may be individually inaccurate, combined together aim to improve performance and reduce over-fitting; finally, SVM classifier has a mathematical approachto the problem because it estimates a hyperplane in the training vector space which optimizes the classification performance.

The performance of the prediction classifiers are evaluated on 100 10-fold cross-validation rounds on the training set of hold-out validation sampling, in terms of Area Under the Curve (AUC) of the Receiver Operating Characteristic (ROC) curve and, once identified the optimal threshold by Youden’s index on ROC curves (30), we have also calculated:

Accuracy=(TP+TN)/(TP+TN+FP+FN),

Sensitivity=TP/(TP+FN),

Specificity=TN/(TN+FP),

where *TP* and *TN* stand for true positive (number of true recurrence cases identified) and true negative (number of true control cases identified), while *FP* (number of control cases identified as recurrence cases) and *FN* (number of recurrence cases identified as control cases) are the false positive and the false negative ones, respectively. Performing 100 rounds of cross-validation allowed having an estimate of the error on the average performance values.

Moreover, the same metrics were used to evaluate the classification performances on the test set of hold-out validation sampling.

## Results

As described above (see section 2.2.1), in order to explore the discriminating power of the features collected from patient medical records, we have implemented three feature importance techniques; in this way we have identified those really useful for the development of an accurate model for predicting the BCR.

### Patients and Cancer Characteristics

Characteristics of the patients are summarized in **Table 1**. A total of 256 patients aged between 24 and 77 (with a median age of 52 years) were included in sample studied. 159 (62%) had no recurrence within 10 years from first diagnosis, whereas, 97 patients (38%) presented recurrence within the first ten years, and among them 54 patients relapsed within 5 years from the first diagnosis.

**Table 1:** Patients characteristics.

Features	With recurrence	Without recurrence
Patients number	97	159
Median age [*q*_1_, *q*_3_]	49 [41, 58]	52 [45, 60]
Histological Subtype		
Ductal	82 (84.54%)	140 (88.05%)
Lobular	5 (5.15%)	12 (7.55%)
Other	2 (2.06%)	2 (1.26%)
Na	8 (8.25%)	7 (4.40%)
Carcinoma *in situ* associated with invasive component		
Absent	47 (48.45%)	81 (50.94%)
Present	32 (32.99%)	52 (32.70%)
Na	18 (18.56%)	26 (16.35*v*)
Vascular invasion		
Absent	35 (36.08%)	91 (57.23%)
Focal	26 (26.80%)	24 (15.09%)
Extensive	13 (13.40%)	4 (2.52%)
Present but not typed	11 (11.34%)	22 (13.84%)
Na	12 (12.37%)	18 (11.32%)
ER		
Median value [*q*_1_, *q*_3_]	70 [20, 90]	45 [10, 80]
Positive (≥ 1%)	79 (81.44%)	123 (77.36%)
Negative (< 1%)	18 (18.56%)	36 (22.64%)
Na	1 (1.37%)	–
PR		
Median value [*q*_1_, *q*_3_]	25 [2, 70]	30 [0, 73]
Positive (≥ 1%)	73 (75.26%)	116 (72.96%)
Negative (< 1%)	22 (22.68%)	43 (27.04%)
Na	2 (2.06%)	–
ki67		
Median value [*q*_1_, *q*_3_]	25 [15, 38]	16 [5, 30]
High (≥ 20%)	61 (62.89%)	81 (50.94%)
Low (< 20%)	34 (35.05%)	77 (48.43%)
Na	2 (2.06%)	1 (0.63%)
Her2		
Positive	19 (19.59%)	31 (19.50%)
Negative	70 (72.16%)	98 (61.64%)
Na	8 (8.25%)	30 (18.87%)
Histological grade		
G1	4 (4.12%)	18 (11.32%)
G2	46 (47.42%)	75 (47.17%)
G3	45 (46.39%)	62 (38.991%)
Na	2 (2.06%)	4 (2.52%)
Tumor size stage		
T1	47 (48.45%)	92 (56.60%)
T2	40 (41.24%)	52 (32.70%)
T3	3 (3.09%)	5 (3.14%)
T4	3 (3.09%)	6 (3.77%)
Na	4 (4.12%)	6 (3.77%)
Lymph nodes stage		
N0	38 (39.18%)	93 (58.49%)
N1	32 (32.99%)	45 (28.30%)
N2	12 (12.37%)	12 (7.55%)
N3	8 (8.25%)	5 (3.14%)
Na	7 (7.22%)	4 (2.25%)
Number of positive lymph nodes		
Median value [*q*_1_, *q*_3_]	1 [0, 5]	0 [0, 2]
Na	3 (3.09%)	2 (1.26%)
Type of surgery		
Quadrantectomy	55 (56.70%)	107 (67.30%)
Mastectomy	42 (43.30%)	52 (32.70%)
Na	–	–
Chemiotherapy	67 (69.07%)	113 (71.07%)
Trastuzumab (Herceptin) therapy	16 (16.49%)	24 (15.09%)
Hormone therapy	79 (81.44%)	124 (77.99%)

For age, ER(%), Pr(%), ki67(%) and number of positive lymph nodes, we have indicated median value and first (*q*_1_) and third (*q*_3_) quarterlies of the distribution.

#### Feature Importance Analysis

We have performed the importance feature analysis on 80% of the sample, that is on 205 patients, considering recurrence both within five and ten years after the first tumor. Specifically, the training set of hold-out validation sampling was made up of 132 cases without recurrence (control cases) and 73 cases with recurrence within ten years after first breast tumor; 41 out of these 73 were recurrences within five years.

**Table 2** shows the feature ranking obtained by different feature importance techniques for predicting BCR within five years after the first diagnosis. The presence of carcinoma *in situ* associated with invasive component was the most informative feature for each of the three techniques developed. NCA technique have identified as more important features also presence of vascular invasion, age, histological grade, tumor size and lymph nodes stage; whereas, the most informative features identified by RF and SVM-RFE techniques were also ki67 and ER hormonal receptors.

**Table 2:** Ranking of the most important features to predict BCR within five years after the first diagnosis of BC obtained by NCA, RF, and SVM-RFE techniques.

Ranking	NCA	RF	SVM-RFE
1	Carcinoma *in situ* associated	Carcinoma *in situ* associated	Lymph nodes stage
	with invasive component	with invasive component	
2	Vascular Invasion	ki67	Tumor size stage
3	Age	ER	Carcinoma *in situ* component
			with invasive component
4	Histological Grade	Vascular invasion	ki67
5	Tumor size stage	Type of Surgery	Histological Grade
6	Lymph nodes stage	Positive lymph node number	ER
7	ki67	Her2	Age
8	Chemiotherapy	Tumor size stage	Chemiotherapy
9	ER	Lymph nodes stage	Vascular Invasion
10	PR	Age	Type of Surgery
11	Type of surgery	Chemiotherapy	Trastuzumab Therapy
12	Positive lymph node number	Trastuzumab therapy	Positive lymph node number
13	Hormone therapy	histological grade	Her2
14	Trastuzumab therapy	Hormone therapy	Histological subtype
15	Histological subtype	PR	Hormone Therapy
16	Her2	Histological subtype	PR

**Table 3** shows the ranking of the most important features to predict BCR within ten years after the first event. The rankings obtained have not changed significantly compared to those identified for the prediction within the 5 years. For this prediction task, respect to previous ranking, NCA technique identified among the most important also type of surgery and HER2; instead, the PR and Vascular Invasion features was more informative for RF and SVM-RFE feature importance techniques, respectively.

**Table 3:** Ranking of the most important features to predict BCR within ten years after the first diagnosis of BC obtained by NCA, RF, and SVM-RFE techniques.

NCA	RF	SVM-RFE	
1	Carcinoma *in situ* associated	Carcinoma *in situ* associated	Lymph nodes stage
	with invasive component	with invasive component	
2	Vascular invasion	Vascular invasion	Carcinoma *in situ* component
			with invasive component
3	Histological grade	ER	Vascular invasion
4	Type of surgery	ki67	Tumor size stage
5	Tumor size stage	Type of Surgery	ER
6	Her2	Her2	Histological grade
7	ER	Positive lymph node number	Age
8	Trastuzumab therapy	PR	Type of surgery
9	Chemiotherapy	Age	Chemiotherapy
10	PR	Tumor size stage	Histological subtype
11	ki67	Lymph nodes stage	Trastuzumab therapy
12	Hormone therapy	Trastuzumab therapy	Her2
13	Age	histological grade	ki67
14	Lymph nodes stage	Histological subtype	Positive lymph node number
15	Positive lymph node number	Chemiotherapy	Hormone therapy
16	Histological subtype	Hormone therapy	PR

### Recurrence Prediction Model

#### Classification performances evaluated on the training set of hold-out validation sampling

In order to identify the optimal features subset useful for accurately predicting the probability of recurrence, we estimated the performances of three classifiers known to the state of the art, such as Naive Bayesian, RF, and SVM on the same 80% of the sample. Specifically, we have trained each classifier on an increasing number of features previously sorted by average importance score calculated by the three techniques described above.

**Figure 2:**
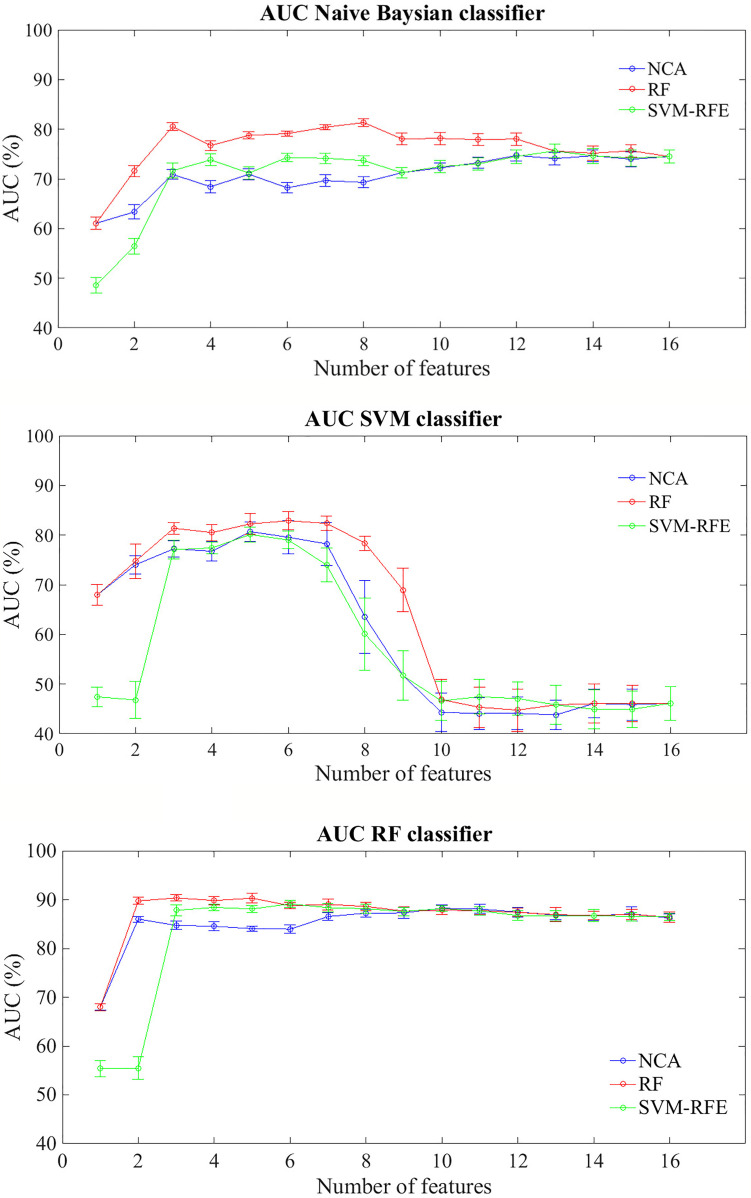
Classification performances for predicting BCR within five years after the first diagnosis for increasing features number sorted by the average importance score. Mean and standard error AUC values evaluated on 100 10-fold cross-validation rounds for each feature importance techniques and classifier.

**Figure 2** shows average AUC values and their standard errors of the BCR classification models within five years after first tumor evaluated on 100 10-fold cross-validation rounds for each feature importance technique

In general terms, RF classifier reached a classification performance of about 68% with the most important feature identified by NCA and RF techniques, i.e. Carcinoma In Situ associated with invasive component; specifically, with the only this features, the classifier showed an average accuracy of 66.40%, a sensitivity of 45.80% and a specificity of 73.54%. Therefore this component does not seem to be able to relapse disease. Indeed, the same classifier showed an average AUC value over around 85% when the features number used to train the model increases, regardless of the method of assessing the importance of the features used. Naive Bayes classifier showed an average AUC value around 71% when the features number selected by the RF technique to train the model was over three; instead, SVM classifier showed an AUC average value around 81% when it trained with a number of features between 3 and 7 selected by RF technique, value beyond which the prediction performances plummet.

In **Table 4** we summarized the classification performances also in terms of accuracy, sensitivity, and specificity in correspondence of the maximum value of average AUC. The best classification performances were obtained by RF classifier; specifically, when it trained on the three features identified as the most informative by SVM technique (i.e. Carcinoma In Situ associated with invasive component, ki67, and ER) the model reached an AUC value of 90.36 ± 0.21%, an accuracy of 77.05 ± 0.94%, a sensitivity of 94.15 ± 1.59%, and a specificity of 71.74 ± 1.70%. Despite, considering the six most important features identified by SVM-RFE technique (i.e. adding lymph Nodes stage, Tumor size stage, histological Grade than those identified by RF technique) the trade off between sensitivity and specificity is reduced (86.59 ± 2.03% and 76.44 ± 2.05%, respectively), without significant reduction of AUC value and accuracy (89.14 ± 0.25% and 78.84 ± 1.10%).

**Table 4:** The best classification performances for predicting the BCR within five years after first diagnosis.

Classifier	Feature set	AUC (%)	Accuracy (%)	Sensitivity (%)	Specificity (%)
	(# selected features)	Mean ± se	Mean ± se	Mean ± se	Mean ± se
Naive Bayesian	NCA (12)	74.72 ± 0.35	72.14 ± 1.13	70.98 ± 1.94	72.50 ± 1.91
	RF (8)	81.34 ± 0.24	74.68 ± 1.50	74.15 ± 2.77	74.85 ± 2.78
	SVM-RFE (13)	75.61 ± 0.42	72.60 ± 1.12	76.34 ± 1.82	71.44 ± 1.77
RF	NCA (10)	88.25 ± 0.21	75.55 ± 0.68	90.44 ± 0.92	70.30 ± 1.10
	RF (3)	90.36 ± 0.21	77.05 ± 0.94	94.15 ± 1.59	71.74 ± 1.70
	SVM-RFE (6)	89.14 ± 0.25	78.84 ± 1.10	86.59 ± 2.03	76.44 ± 2.05
SVM	NCA (5)	80.69 ± 0.61	84.22 ± 0.69	70.00 ± 1.46	88.64 ± 1.11
	RF (6)	82.89 ± 0.57	85.84 ± 0.64	73.90 ± 0.89	89.55 ± 0.92
	SVM-RFE (5)	80.10 ± 0.48	83.18 ± 0.91	67.56 ± 2.70	88.03 ± 1.97

Mean and standard error (se) of AUC values, Accuracy, Sensitivity, and Specificity evaluated on 100 10-fold cross-validation rounds for each feature importance technique and classifier.

It should be underlined that SVM classifier trained on the six features identified as more important by RF technique (i.e. the same 3 features above indicated and moreover type of Surgery, Number of positive lymph node involvement and Her2) showed a higher accuracy (85.84 ± 0.64%) but a lower sensitivity (73.90 ± 0.89% i.e. a lower probability to identify recurring cases.

**Figure 3:**
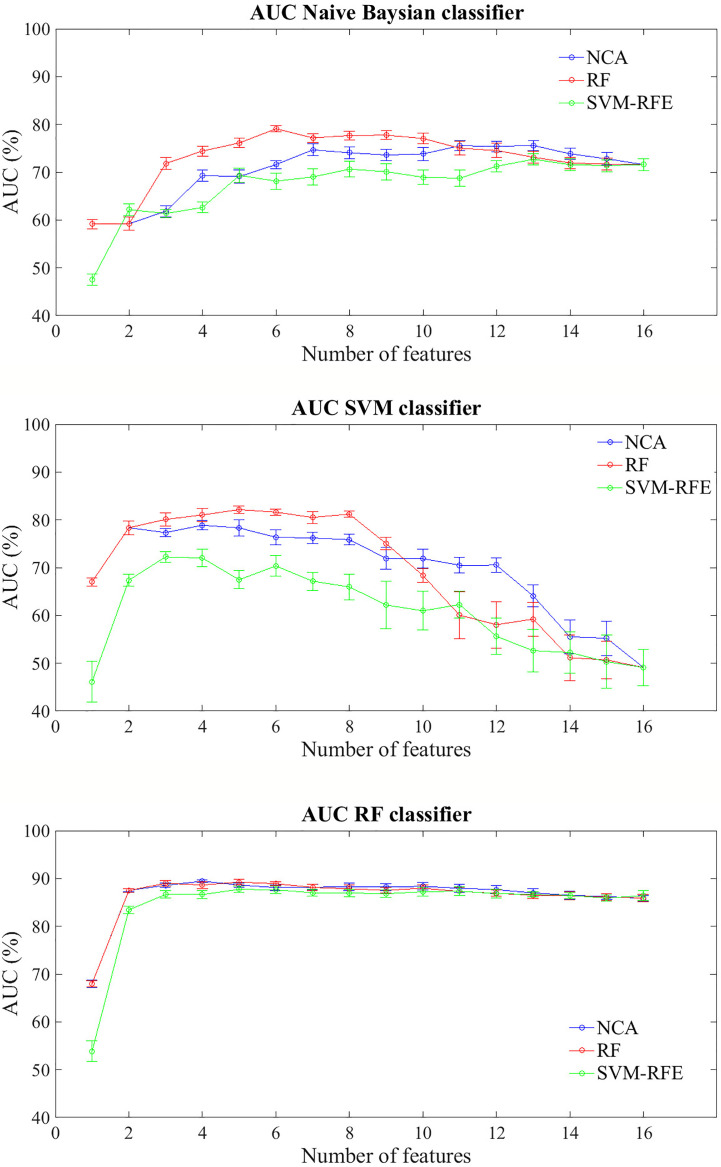
Classification performances for predicting BCR within ten years after the first diagnosis for increasing features number sorted by the average importance score. Mean and standard error AUC values evaluated on 100 10-fold cross-validation rounds for each feature importance techniques and classifier.

**Figure 3** shows the prediction performances of the BCR within ten years after first tumor for each feature importance technique and classifier model trained. The trend of the three classifiers trained on increasing features number sorted by average importance score calculated with the three techniques described above, follows a similar trend to that observed for the prediction problem within five years. Similarly to the prediction model of recurrence within five years, RF classifier still showed classification performance and reached an AUC value of about 68% with a single feature (Carcinoma In Situ associated with invasive component) identified as best feature by NCA and RF techniques; moreover, it showed an average AUC value over 86% as the number of features used to train the model increases, regardless of the method of assessing the importance of the features used. Naive Bayes classifier showed an average AUC value around 73% when the feature number used to train model by using the subset features selected by NCA or RF techniques was over four, whereas SVM classifier showed an average value of AUC around 81% when it is trained with a number of features between 2 and 8 selected by RF technique.

The best classification performances in terms of average AUC value are obtained by RF classifier. Specifically, with the four most important feature identified by NCA technique (i.e. Carcinoma In Situ associated with invasive component, Vascular Invasion, histological Grade, and type of Surgery) the model reached an AUC value of 89.45 ± 0.10%, an accuracy of 83.66 ± 0.68%, a sensitivity of 70.68 ± 1.84%, and a specificity of 90.83 ± 0.02% (**Table 5**). With the same classifier when trained with the five most important features identified by the RF or SVM-RFE techniques, the sensitivity raised to about 95% but the specificity fell to 67%. It should be underlined that SVM classifier trained on the five features identified as more important by RF technique (i.e. Carcinoma In Situ associated with invasive component, Vascular Invasion, ER, ki67, and type of Surgery) showed a best trade-off between sensitivity and specificity (77.67 ± 0.58% and 87.73 ± 0.57%, respectively), with an accuracy of 84.15 ± 0.29%.

**Table 5:** The best classification performances for predicting the BCR within ten years after first diagnosis.

Classifier	Feature set	AUC (%)	Accuracy (%)	Sensitivity (%)	Specificity (%)
	(# selected features)	Mean ± se	Mean ± se	Mean ± se	Mean ± se
Naive Bayesian	NCA (13)	75.59 ± 0.34	68.49 ± 0.18	92.47 ± 0.23	55.23 ± 0.29
	RF (6)	79.10 ± 0.21	69.90 ± 0.15	96.30 ± 0.29	55.30 ± 0.23
	SVM-RFE (13)	72.68 ± 0.36	67.80 ± 0.60	89.86 ± 1.05	55.61 ± 1.37
RF	NCA (4)	89.45 ± 0.10	83.66 ± 0.68	70.68 ± 1.84	90.83 ± 2.02
	RF (5)	89.25 ± 0.18	77.17 ± 0.48	95.34 ± 0.89	67.12 ± 1.15
	SVM-RFE (5)	87.73 ± 0.21	77.51 ± 0.25	95.48 ± 0.84	67.58 ± 0.61
SVM	NCA (4)	78.87 ± 0.32	84.63 ± 0.28	69.18 ± 0.51	93.18 ± 0.51
	RF (5)	82.14 ± 0.24	84.15 ± 0.29	77.67 ± 0.58	87.73 ± 0.57
	SVM-RFE (3)	72.25 ± 0.35	76.73 ± 0.79	65.89 ± 1.05	82.73 ± 1.58

Mean and standard error (se) of AUC value, Accuracy, Sensitivity, and Specificity evaluated on 100 10-fold cross-validation rounds for each feature importance technique and classifier.

#### Classification Performances Evaluated on the test set of Hold-Out Validation Sampling

On the training set of hold-out validation sampling, we have found the best prediction model learning to five years follow-up reached an AUC value, accuracy, sensitivity and specificity of 89.14 ± 0.25%, 78.84 ± 1.10%, 86.59 ± 2.03%, and 76.44 ± 0.05%, respectively, with only six features, that is Carcinoma In Situ associated with invasive component, ki67, ER, lymph Nodes stage, Tumor size stage, and histological Grade selected by SVM-RFE technique and RF classifier. Indeed, the best prediction model learning to 10 years follow-up reached an AUC value of 89.25 ± 018%, an accuracy of 77.17 ± 0.48%, a sensitivity of 95.34 ± 89%, and a specificity of 67.12 ± 1.15% with only five features, i.e. Carcinoma In Situ associated with invasive component, Vascular Invasion, ER, ki67 and type of Surgery selected by RF technique an classifier.

**Figure 4:**
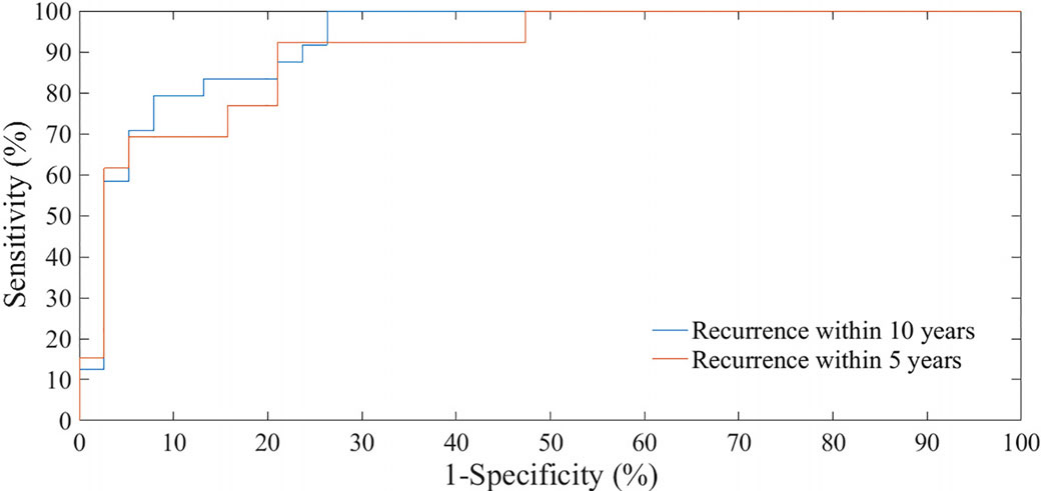
ROC curves of classification performances for predicting BCR within five and ten years after the first diagnosis evaluated on test set of hold-out sampling.

We have evaluated the classification performances of the best models on 20% of the sample, that is on 51 patients, both with reference to recurrence within five years tumor and within ten years after the first tumor (**Figure 4**). The test set of holdout validation sampling was made up of 27 cases without recurrence (control cases) and 24 cases with recurrence within ten years after first breast tumor. 13 out of these 24 were recurrences within five years. On the test set of holdout validation sampling, the classification model learning to 5 years follow-up reached an AUC value, accuracy, sensitivity and specificity of 87.75%, 77.50%. 92.31%, and 70.37%, respectively, whereas the performances of the model learning to 10 years follow-up were 91.20%, 80.39%, 95.83%, and 66.67% (**Table 6**).

**Table 6:** The classification performances for predicting the BCR within five and ten years after the first diagnosis evaluated on test set of hold-out sampling.

Follow-up	AUC (%)	Accuracy (%)	Sensitivity (%)	Specificity (%)
5 years	87.75%	77.50%	92.31%	70.37%
10 years	91.20%	80.39%	95.83%	66.67%

## Discussion

The goals of this work is to evaluate the prognostic power, histopathologic, and therapeutic patient characteristics in order to develop a machine learning model to predict BCR.

In this our preliminary work, firstly we implemented several feature importance techniques and then we evaluated the prediction performances of BCR within 5 and 10 years after the first diagnosis by means different classifiers at the state-of-the-art.

The standard duration of adjuvant endocrine treatment has been 5 years for a long time. Nevertheless, based on the evidence of data reported in the literature, the extension of hormone therapy up to 10 years should be evaluated (31, 32). Therefore, a purpose of our research is also to predict patients with high probabilities of late relapse to adjust individual follow-up programs and the long-term hormonal treatment.

Our experimental results showed that RF classifier have reached high performances both with reference to the disease recurrence prediction model within 5 years and within 10 years. As mentioned above, RF classifier is an ensemble model i.e. it provides a classification on the majority result with respect to singularly trained trees generating a more accurate classification than mathematical or probabilistic models, which need more stringent parametric requirements.

Specifically, on the test set of hould-out validation sampling, the prediction model learning to 5 years follow-up reached an AUC value, accuracy, sensitivity and specificity of 87.75%, 77.50%. 92.31%, and 70.37%, respectively, with only six features, that is Carcinoma In Situ associated with invasive component, ki67, ER, lymph Nodes stage, Tumor size stage, and histological Grade selected by SVM-RFE technique.

Indeed, the prediction model learning to 10 years follow-up reached an AUC value of 91.20%, an accuracy of 80.39%, a sensitivity of 95.83%, and a specificity of 66.67% with only five features, i.e. Carcinoma In Situ associated with invasive component, Vascular Invasion, ER, ki67 and type of Surgery selected by RF technique.

As proved in several studies, tumor size stage, vascular invasion, histologic Grade, lymph Nodes stage are associated with a higher risk of recurrence (33, 34). It difficult to define a threshold value tumor size below or above which the tumor can be considered as having a bad or good prognosis, except for very small tumors, and the risk assessment cannot be separated from considering the other prognostic parameters. The results of the MIRROR study showed that also the presence of isolated cells or micro-metastases in the regional lymph nodes is associated, in the absence of adjuvant therapies, with a worse disease-free survival (35). A high histological grade (G3) is considered an unfavorable prognostic factor (36).

Proliferative activity measured with the Ki67 labeling index (percentage of tumor cell nuclei that stain with the antibody for the Ki67 protein encoded by the KI67 gene) is now a recognized prognostic indicator of increased cancer recurrence (37). Moreover, some studies have shown its prognostic value and its usefulness in predicting the response and clinical outcome (38). The new ASCO recommendations for the immunohistochemical determination of ER and PR hormone receptors consider tumors with at least 1% of positive cells to be positive (39).There is a relationship between the levels of positive receptors and the benefits obtained with hormonal treatments, i.e. tumors with high levels of receptors are those that are more likely to benefit from hormone therapy. Moreover, patients with ER-positive BC maintain a significant recurrence rate during extended follow-up (40).

Several studies have shown vascular invasion (defined as the presence of clear signs of invasion in at least ten microscopic fields) is predictive of worse shooting-free survival and overall survival in patients with other risk factors such as histological grade, tumor size and hormone receptor status (41, 42).

Conversely, Ductal Carcinoma In Situ associated with invasive carcinoma is not universally accepted as a prognostic factors. Ductal Carcinoma In Situ associated with invasive carcinoma is characterized by proliferation of ductal epithelial cells confined within the ductal-lobular system and is a potential precursor of invasive BC. Indeed, while Ductal Carcinoma In Situ is not life threatening, it does increase a woman’s risk of developing invasive BC later in life, which subsequently could lead to a BC-specific death. In a few studies, investigators showed that various histopathological characteristics of Ductal Carcinoma InSitu, such as lesion size, marginal status, histological grade, architectural patterns, and presence of necrosis were associated with recurrence. The size of the Ductal Carcinoma In Situ component after conservative breast surgery and radiotherapy can increase the risk of recurrence as well as the positivity of surgical resection margins and the presence of unfavorable histopathological features (high grading, comedonecrosis in Carcinoma In Situ). Mastectomy in the presence of extensive intraductal component is therefore indicated (43, 44).

**Table 7** summarizes the results of the proposed method and the main state-of-the-art works for predicting BCR. In general terms, most of the works proposed in literature have developed prediction models of disease recurrence within 5 years. Variables used in the considered studies were mainly of three types, that are patient characteristics, cytological characteristics, and treatments, and only one used also textural ones. We have considered works that used both public and private database; since all the databases collect heterogeneous data, often not coming from the same research centers, this comparison is justified. Finally, as these works used different databases and classifier models, the comparison is purely qualitative.

**Table 7:** Performance comparison of the proposed models with respect to the literature.

Papers	Overall dataset	Features	Methods	Sampling	Best
	(recurrence/no recurrence)			strategy	performances (%)
Mohebian et al. (8)	Private database	Patient	Particle Swarm	Hold-out	AUC: 90.0%
	(112/467)	and histopathological	Optimization		Acc: 90.0%
		characteristics,	and Bagged		Sens: 81.0%
	Follow-up 5 year	and therapy	Decision Tree		Spec: 98.0%
Beheshti et al. (45)	Wisconsin Prognostic BC	Textural	Genetic	Hold-out	AUC: 63.0%
	(UCI Repository)	and histopathological	Programming		Acc: 80.3%
	(47/151)	characteristics	approach		Sens: 52.3%
	Follow-up 5 year				Spec: 83.4%
Chaurasia and Pal (9)	University Medical Centre,	Patient	Simple Logistic	10-fold	AUC: -%
	Ljubljana, Yugoslavia	and histopathological		cross validation	Acc: 74.4%
	(85/201)	characteristics,			Sens: 31.8%
	Follow-up 5 year	and therapy			Spec: 92.5%
Kim et al. (11)	Private database	Histopathological	SVM	Hold-out	AUC: 85.0
	(195/484)	characteristics			Acc: 84.6%
		and therapy			Sens: 89.0%
	Follow-up 5 year				Spec: 73.0%
Proposed models	Private Database	Patient	SVM-RFE feature	10-fold	AUC: 89.1%
	(54/159)	and histopathological	selection and	cross-validation	Acc: 78.8%
		characteristics,	RF classifier		Sens: 86.6%
	Follow-up 5 years	and therapy			Spec: 76.4%
Proposed models	Private database	Patient	SVM-RFE feature	Hold-out	AUC: 87.8%
	(54/159)	and histopathological	selection and		Acc: 77.5%
		characteristics,	RF classifier		Sens: 92.3%
	Follow-up 5 years	and therapy			Spec: 70.4%

The performances of our model was high performing with respect to considered works. The models proposed in Chaurasia and Pal (9) and Beheshti et al. (45) have shown a low probability of predicting disease recurrence. Specifically, Chaurasia et al. Chaurasia and Pal (9) used a sample numerically comparable with ours and reached a sensitivity of about 32% by using a parametric statistical model. Kim et al. (11) used hold-out validation method (70% training set; 30% test set) and reported the algorithm performances on the test set comparable with our results with the same sampling strategy. Mohebian et al. Mohebian et al. (8) had identified as age at diagnosis, tumor size, lymph node involvement ratio (defined as ratio of involved to dissected lymph nodes), number of involved axillary lymph nodes, PR, hormone therapy, and type of surgery, as more important factors to predict BCR within 5 years. Their model evaluated by using hold-out validation method (70% training set; 30% test set) have reached an overall accuracy of 90.0% but a sensitivity of 81.0%. Compared to Mohebian et al. (8), Kim et al. (11), our models showed a higher sensitivity (92.3%), i.e. a higher predictive accuracy of patients who will experience a relapse of disease in the short term.

A limit of our work was the presence of missing data which can introduce bias and reduce efficiency (14). However, this situation occurs in real applications, therefore we preferred not to eliminate the subjects with the missing data, but to estimate such incomplete data. Moreover, it should be emphasized that for the definition of a decision support system useful in clinical practice, it is necessary to evaluate this tool on a necessarily larger and more representative sample of patients with breast cancer. Nevertheless, our preliminary results are encouraging and the sample studied is numerically comparable with other studies at the state of the art as indicated in **Table 7**. Although the model reaches a relevant level of accuracy, specificity, i.e. the forecast of patients who will not relapse within 5 or 10 years is not sufficiently high for the use of this system in clinical practice. In fact, this performance value would compromise the efficiency of this tool in the most appropriate therapeutic choice, suggesting a more aggresive therapeutic treatments and probably less appropriate. Therefore in future studies it is necessary to improve prediction performances also by evaluating the introduction of new features of a different kind, such as for example radiomic ones extracted from images of first level instrumental exams (mammograms or ultrasounds), and optimizing the models will be optimized through an appropriate parameter tuning study.

After an important and systematic evaluation phase of the model on a larger data set, such automated support tool might be used by clinicians to significantly extend patient life (12). Specifically, it could be useto define an appropriate treatment strategy by suggesting to the multidiscipinary team (i.e. breast radiologists, surgeons, and medical and radiation oncologists) the most promising therapeutic treatment (surgery, and chemio, hormone or biological therapy) or the combination of them in terms of probabilities of recurrence free-survival at 5 or 10 years (12). This tool, in association with the expertise of professional figures, would have a significant impact on clinical activity due to the real-time availability of a probable prognostic result objectively evaluated on the retrospective data and to the best interpretation of the latter. The containment of any subjective assessment errors would lead to an invaluable improvement in the quality of life of patients and the general satisfaction of professionals.

## Conclusions

In this work we presented preliminary results of a feature importance analysis collected by patient records aimed to develop of an automated system to predict the BCR. Although different approaches have been used in the literature, it is still an open task which includes the collecting of suitable and quality data-set, the defining of a proper features selection procedure and the training of an accurate prediction model. Despite occasionally BC patients relapsed after more than 5 years after initial treatment and sometimes also with highly aggressive disease, most of the works proposed in literature has developed prediction models of disease recurrence within 5 years. In our work, we also obtained encouraging results for the forecast of the recurrence of disease in the long term, that is, beyond 5 years of follow-up.

Indeed, our proposed models for predicting BCR within 5 and 10 years after diagnosis showed highly performances using a small number of features. Specifically, our experimental results showed a high prediction sensitivity, i.e. the probability to predict disease recurrence. This represents an important requirement for a reliable support prognostic tool because it provides a useful indication for clinicians for defining a personalized treatment plan.

The most important feature was Carcinoma In Situ associated with invasive component both to predict no recurrence within five and ten years. Indeed, although it is not yet universally accepted as a prognostic factor, recent studies have shown invasive Ductal Carcinoma accompanied by Ductal Carcinoma In Situ was associated with lower local recurrence (46). The prognostic value of Ductal Carcinoma In Situ component in decision-making process could be considered as a new independent prognostic marker (47). Future works include validation studies for testing the robustness of the obtained results in a larger population that will be framed in a multi-centre research project funding by Ministry of Health. In order to improve the diagnostic accuracy, the model will be optimized by means appropriate parameter tuning study. Moreover, we will evaluate the usefulness of radiomic features in BCR prediction and the contribution of other types of features with respect to those used in this work. Indeed, despite some studies show their predictive prognostic power, in literature there are few studies using them, whereas the most of them concern the development of computer aided diagnosis systems for the cancers characterization (48–53).

## Data Availability Statement

The raw data supporting the conclusions of this article will be made available by the authors, without undue reservation.

## Ethics Statement

Ethical review and approval were not required for the study on human participants in accordance with the local legislation and institutional requirements. Written informed consent for participation was not required for this study in accordance with the national legislation and the institutional requirements.

## Author Contributions

Conceptualization, RM, AF, AL, and VL. Methodology, RM and AF. Software, AF. Validation, AF. Formal analysis, RM and AF. Data curation, RM, AF, AL, AR, and VL. Writing–original draft preparation, RM, AF, AL, and VL. Writing–review and editing, RM, AL, AF, RB, VD, FG, DL, AN, MP, CR, LR, AR, PT, ST, AZ, and VL. Supervision, RM, AL, and VL. All authors have read and agreed to the published version of the manuscript. All authors contributed to the article and approved the submitted version.

## Funding

This work was supported by funding from the Italian Ministry of Health “Ricerca Corrente 2018–2020”.

## Conflict of Interest

The authors declare that the research was conducted in the absence of any commercial or financial relationships that could be construed as a potential conflict of interest.
